# A comparison of the effects of electronic reminders and group bonuses on the recording of diagnoses in primary care: a longitudinal follow-up study

**DOI:** 10.1186/s13104-017-3054-2

**Published:** 2017-12-06

**Authors:** Tuomo Lehtovuori, Marko Raina, Lasse Suominen, Timo Kauppila

**Affiliations:** 1 Siltakatu 11, 3. krs, 02770 Espoo, Finland; 2 Peltolantie 2, 01300 Vantaa, Finland; 3 Department of General Practice and Primary Health Care, HUS, Institute of Clinical Medicine, University of Helsinki, P.O. Box 20, Tukholmankatu 8 B, 00014 Helsinki, Finland

**Keywords:** Community health centers, Medical informatics, Primary care, Practice management, Quality improvement

## Abstract

**Objective:**

To improve the recording of diagnoses in visits to general practitioners, an observational retrospective study based on a before-after design was performed by installing an electronic reminder in the computerized patient chart system, reinforced in feedback delivered in superior-subordinate or development discussions with the general practitioners. The monthly rate of recording diagnoses was observed before and after the intervention. The effect of this intervention on recording of diagnoses was compared with the effects of financial group bonuses on the same parameter in a neighbouring city.

**Results:**

Before intervention, the level of recording diagnoses was about 45% in the primary care units. Nine months after this intervention there was not yet any statistically significant increase in recording of diagnoses but after 21 months it yielded a recording rate of 90% (P < 0.001). In three years, this percentage reached level over 95%. Group bonuses, a financial incentive serving as a control intervention, increased this parameter from 50 to 80% (P < 0.001) in nine months, and in 21 months the level of recording diagnoses was 90%. The both methods increased the level of recording diagnoses at the same level. Group bonuses acted faster but were also more expensive.

## Introduction

In primary care, the recording of diagnoses is needed to ensure sufficient treatment actions, planning activities and management of resources [1–3]. In primary care, financial incentives to individual GPs [4] or to multidisciplinary care teams [5] have been reported to be effective in increasing the recording of diagnoses.

In the primary care of Vantaa, the basic frequency of recording disease diagnoses was about 45%, which was considered insufficient. A higher frequency of recorded diagnoses was required for planning activities and managing the resources. In a quite similar neighboring city, Espoo, it was possible to increase the frequency of recording diagnoses from 55% of all visits to general practitioners to a level of 90% by using financial group bonuses for primary care teams [5]. Vantaa had no resources for such financial incentives. Since electronic reminders have also been shown to be effective in modifying the work practices of GPs [6] the administration of Vantaa primary care installed an electronic reminder into the computerized patient chart system to improve the recording of diagnoses by the care teams in one of its regions, Hakunila-Länsimäki. This intervention was enhanced in feedback delivered in superior-subordinate or development discussions with the GPs.

The aim of this study was to discover whether the electronic reminders of the patient chart system, enhanced with feed-back discussions, increased the rate of recording disease diagnoses following GPs’ visits. We also wanted to explore whether this effect was in any way different from the effect of group bonuses, a financial incentive which had proved to be successful in increasing diagnosis recordings [5].

## Main text

### Methods

#### Study design

The present work is a retrospective longitudinal quasi-experimental study with a before- and after-design in the primary care of the second largest city of Finland. This study was performed in Hakunila-Länsimäki, a region of Vantaa city, where in 2008 there were about 23,000 inhabitants (total number 200,000 in Vantaa city, 2008). In Finland, primary care is non-profit and municipalities maintain and fund this activity with taxes. The number of doctors varied from 12 to 15 during the follow-up period.

#### Study measures and outcomes

The data of the combined Länsimäki-Hakunila health center were gathered from Graphic Finstar patient chart system (GFS, Logica LTD, Helsinki, Finland). The report generator of the Finstar-system provided the total number of GP visits, the number (but not quality) of recorded diagnoses and a percentage for the recording of diagnoses for each individual GP. This allowed the calculation of a mean of these percentages, the main measure for analysis in the present study. To get reliable data from GFS the report generator requires precise pre-identification of the doctor under study at a given time and it is therefore not able to produce continuous monthly data throughout the whole system. Therefore, the busiest month of the year (November [7]) was chosen as the control data and comparison between the controls was performed by using this single period. In February 2008, an electronic reminder was installed into GFS. If the doctor did not mark a diagnosis to the patient chart, the computer asked at the end of the report “Are you going to finish the report without marking the diagnosis?” The doctor had then a possibility to close the report by answering “yes”. If the doctor answered “no” the patient chart system returned automatically back to the appropriate place to mark the diagnosis. If the diagnosis was then recorded, the patient chart system allowed finishing the report without any further enquiries. The effect of the electronic reminder was enhanced in feedback delivered in superior-subordinate or development discussions with the GPs. These discussions took place once a year and they lasted 45 min^–1^ h. The follow-up period started from 2002 to ended 2012.

As a control, we used data from primary care of Espoo where group bonuses were applied in March 2005 [5]. Espoo resembles Vantaa in its location (neighboring Helsinki) and number of inhabitants (about 230,000) and also in other factors such as age, sex, morbidity levels, deprivation and other demographic factors (see http://www.aluesarjat.fi, and http://pxweb2.stat.fi/database/StatFin/databasetree_fi.asp). Therefore, we [5, 7] and others [8] have used Espoo and Vantaa as control cities to each other in former studies. Analogous data from November 2003 were obtainable from Espoo primary health care (Effica patient chart system, Tieto LTD, Helsinki, Finland). Both systems, Effica and Finstar, gave a similar specific place in the electronic patient chart where appropriate ICPC-2 or ICD-10 diagnoses could be placed during the patient visit. Both systems assisted the GP to find a proper diagnosis code or allowed the doctor to use directly the right code for the desired diagnosis. No ethical approval was required because this study was made directly from the patient registry without identifying the patients or diagnoses. The registry keepers (the health authorities of Espoo and Vantaa) granted permission to carry on the study.

#### Statistical analysis

The obtained data were analyzed by comparing the recording of diagnoses during similar periods before and after the installation of the electronic reminder into the patient chart system of primary health care in Hakunila-Länsimäki. The variation within a single care team in Länsimäki-Hakunila primary care was analyzed by using the mean care unit-based percentage of monthly doctor visits with recorded diagnoses over the whole study period. The comparisons were performed by using non-parametric One Way Repeated Measures ANOVA (RM-ANOVA) with suitable corrections (Bonferroni) for multiple comparisons when following the development of the studied units as a function of time. When respective time periods after and before the two different interventions were compared, t test was applied. The rate of change in diagnosis marking was analyzed using regression analysis followed by t test (GLM procedure of SigmaPlot 10.0 Statistical Software, Systat Software Inc., Richmond, CA, USA) [5].

## Results

### Effect of electronic reminder

**Fig. 1:**
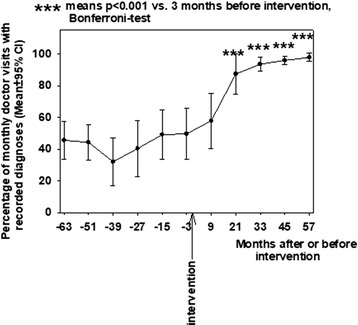
Effect of electronic reminders on the team-based percentage of monthly doctor visits with recorded diagnoses in Hakunila-Länsimäki primary care 2002–2012. Percentage of GPs’ visits with recorded diagnoses are presented before and after introducing electronic reminder in February 2008. Means (dots) and 95% CI (brackets) are shown

The rate of change in the recording of diagnoses was 2.3 ± 2.1%/year (mean ± SEM) in 2002–2007, e.g. before the intervention. After the intervention, this rate increased to 11.6 ± 1. 8%/year (P < 0.001, t test). The percentage of recording diagnoses in the units was statistically significantly higher 21, 33, 45 and 57 months after the application of electronic reminders when compared with the pre-intervention level (P < 0.001, RM-ANOVA, Fig. 1).

### Comparison with group bonuses

**Fig. 2:**
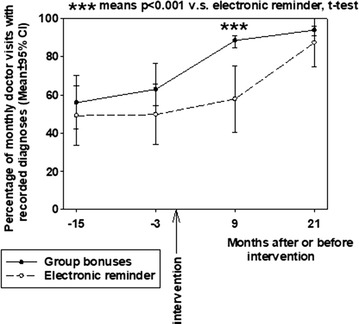
Comparison between the effects of group bonuses and electronic reminders on the team-based percentage of monthly doctor visits with recorded diagnoses in primary care. Percentage of GPs’ visits with recorded diagnoses are presented before and after introducing electronic reminder or group bonuses. Means (dots) and 95% CI (brackets) are shown

Group bonuses increased recording of diagnoses too (P < 0.001, RM-ANOVA, Fig. 2). The 21-month level of recorded diagnoses using electronic reminders was achieved after only 9 months by using group bonuses. The level of recorded diagnoses after 9 months using electronic reminders was significantly lower than the level after a similar period using financial incentives (Fig. 2).

## Discussion

An electronic reminder with superior-subordinate or development discussions increased the rate of recording diagnoses in the visits to GPs. The diagnoses were recorded in more than 95% of the visits to GPs within 3 years after beginning of this intervention, which was more than that eventually reached with financial group incentives [5]. One strength of this study is that the present retrospective setting led to a situation where the study objects did not know that they were especially studied. Furthermore, we got this data from all visits to the GPs. Thus, the present result reflects real clinical activity.

Electronic reminders have been shown to be effective in modifying the work of GPs [6] but as far as we know it has never been reported that they have been used for the same purpose as in the present study. Neither have there been reports comparing the effects of electronic reminders with financial incentives which are known to be useful in altering the behavior of primary care doctors, too [5, 9, 10]. At the end of the follow-up period the level of recording diagnoses was practically the same in both the electronic reminder and team bonus groups. This result is in line with a former study suggesting that the commitment of the staff is equally important as financial incentives when improving the quality of clinical work [11].

Electronic reminders were thus effective in altering the behavior of GPs as expected [6]. When combined with superior-subordinate or development discussions it led to the same level in recording of diagnoses as group bonuses. The group bonuses were faster in improving the recording rate of diagnoses than the electronic reminder. However, the costs of group bonuses (over 100.000 €/each year in the present case) [5] are considerably higher than using electronic reminders.

## Conclusions

Because the final level of recorded diagnoses was at the same level after both types of intervention, electronic reminders with feedback delivered in superior-subordinate or development discussions with the GPs can be considered as a more preferable method to enhance this activity than financial incentives.

## Limitations

- A limitation of this study is that the present results cannot be directly extrapolated to other sections health care than primary care.
- Although we did not identify or account for other, secular trends operating concurrently to the intervention we cannot totally exclude existence of such trends in the present experimental setting.
- The scientists were not consulted by the administration when the intervention was performed and therefore the retrospective study was the only possible setting. This led to a situation where data about other, putatively equally interesting, parameters such as distribution of recorded diagnoses in visits to GPs were not collected.

## Abbreviation

GP: general practitioner
